# Investigating Mining-Induced Surface Subsidence in Mountainous Areas Using Integrated InSAR and GNSS Monitoring

**DOI:** 10.3390/s26041222

**Published:** 2026-02-13

**Authors:** Qingfeng Hu, Runjin Hou, Yingchao Kou, Peng Wang, Zilin Liu, Huaizhan Li, Wenkai Liu, Xinjing Wang, Sihai Yi, Fan Zhang, Zhaomeng Zhou, Mingyang Zhang, Xinlei Li, Qifan Wu

**Affiliations:** 1 College of Surveying and Geo-Informatics, North China University of Water Resources and Electric Power, Zhengzhou 450046, China; z20241151131@stu.ncwu.edu.cn (R.H.); z20211151045@stu.ncwu.edu.cn (P.W.); z20231151103@stu.ncwu.edu.cn (Z.L.); liuwenkai@ncwu.edu.cn (W.L.); wangxinjing@ncwu.edu.cn (X.W.); z20241151143@stu.ncwu.edu.cn (F.Z.); z20241151133@stu.ncwu.edu.cn (Z.Z.); z20241151130@stu.ncwu.edu.cn (M.Z.); wuqifan@ncwu.edu.cn (Q.W.); 2 Zhengzhou Planning, Survey, Design and Research Institute Co., Ltd., Zhengzhou 450000, China; kyc0619@163.com; 3 Engineering Research Center of Ministry of Education for Mine Ecological Restoration, China University of Mining and Technology, Xuzhou 221116, China; lihuaizhan@cumt.edu.cn; 4 School of Safety Engineering, North China Institute of Science and Technology, Beijing 101601, China; tsyisihai@163.com; 5 Jiaozuo Coal Industry (Group) Xinxiang Energy Co., Ltd., Xinxiang 453000, China; lixinlei99@163.com

**Keywords:** InSAR, GNSS, PPP, mining-induced subsidence, mining area monitoring

## Abstract

Leveraging the complementary advantages of InSAR and GNSS, this study proposes a refined method for monitoring mining-induced surface subsidence by integrating both technologies. The method begins with calculating the time-series cumulative subsidence basin from InSAR. Subsequently, a constraint condition is established to identify large-gradient deformations, thereby distinguishing the subsidence edge from the subsidence center. For the subsidence edge with minor deformation, the InSAR results are retained. For the large-gradient subsidence center, the subsidence basin around the mining panel is reconstructed by integrating InSAR and GNSS models. Continuous surface deformation information in a geographic coordinate system is then obtained through spatial interpolation, ultimately yielding comprehensive surface subsidence results across the mining area. Taking a mining area in Shanxi Province as the study region, the feasibility and accuracy of the proposed method were validated using 35 SAR images acquired between April 2016 and September 2017, along with leveling measurement data from the mining panel. The maximum surface subsidence rate of the settlement basin obtained from the solution is −186.68 mm/year, and the maximum surface subsidence amount is 248 mm. Compared with the InSAR monitoring results, the root mean square error of the data collaborative monitoring is reduced by 96.8%, and it is reduced by 64.4% compared with the GNSS probability integral method. The results demonstrate that the proposed method can achieve subsidence results consistent with the actual situation. Its monitoring capability is significantly superior to that of using either InSAR or GNSS alone, effectively compensating for the limitations inherent in each individual technology when applied to mining subsidence monitoring. Consequently, this integrated approach provides more accurate and reliable information on surface subsidence in mining areas.

## 1. Introduction

China is the world’s largest coal producer [1], with over 95% of its coal resources extracted by longwall mining [2]. By the end of 2017, coal mining had induced subsidence areas covering approximately 20,000 square kilometers [3]. Nevertheless, coal remains a foundational strategic resource and continues to dominate the primary energy production and consumption of China, with no indication that this dominance will wane. It is projected that coal will still constitute around 50% of the primary energy mix by 2050 [4]. To meet the demands of national economic development, large-scale extraction of coal resources will continue [5]. The intensive, large-scale mining of underground coal seams has made significant contributions to China’s economic development and societal progress. Simultaneously, it has triggered severe environmental and geohazard issues in mining regions, including ground subsidence, structural damage, water resource contamination, and landslides [6,7,8,9]. These phenomena pose substantial risks to the lives and property of local residents. Approximately one-third of China’s coal mines are located in mountainous areas. Due to the complex topography and geomorphology, the impact of coal mining on the surface is far more pronounced than in the plains. Surface subsidence caused by mining in mountainous areas results in greater hazards and losses [10,11,12]. Consequently, the development of effective, precise, and comprehensive monitoring methods for mining-induced surface subsidence has become a critical yet challenging focus in current research on mountainous mining areas. Traditional monitoring techniques for surface subsidence in such regions primarily include total station surveying, leveling, and theodolite measurements. However, these methods suffer from high labor intensity, elevated costs, and limited spatial coverage. They are often constrained by environmental and temporal factors, failing to accurately characterize the overall spatial pattern and temporal trend of ground subsidence across mining areas [13,14]. To gain an in-depth understanding of surface deformation patterns in mountainous mining regions, it is imperative to employ novel technologies for comprehensive, high-precision monitoring. Traditional Global Navigation Satellite System (GNSS) surveying can precisely measure three-dimensional deformation at specific points [15,16]. However, its reliance on a network of reference stations presents limitations, including sparse measurement points, suboptimal network geometry, and complex data processing. These constraints make it difficult to capture detailed subsidence information at the edges of mining areas. Precise Point Positioning (PPP) is an absolute positioning data processing method based on precise orbit and clock products that integrates the advantages of both standard GNSS point positioning and relative positioning [17,18]. Utilizing only a single receiver, PPP can obtain high-precision position coordinates without distance limitations, offering both flexibility and cost-effectiveness. This effectively mitigates the traditional GNSS shortcomings related to point density and the acquisition of edge deformation information [19,20]. Research by Jin et al. demonstrated the significant role of GNSS PPP technology in geodetic surveying, time-frequency transfer, and disaster monitoring and early warning [21]. Kudlacik et al. utilized PPP technology with a dense network of low-cost GNSS receivers in a mining area to acquire deformation data, enabling a more refined analysis of post-mining ground deformation and yielding more accurate deformation parameters for the copper mining area [22]. Interferometric Synthetic Aperture Radar (InSAR) technology has been widely applied in ground subsidence monitoring due to its advantages of broad coverage, all-weather capability, and high precision [23,24,25]. Differential Interferometric Synthetic Aperture Radar (D-InSAR), which employs differential interferometry, imposes stringent requirements on the coherence of master and slave images. The large deformation gradients often encountered in mining areas can exceed the phase unwrapping threshold, leading to unwrapping failures and inaccurate deformation retrieval [26]. Shi et al. introduced boundary constraints when determining the integration interval for SBAS-InSAR and the Probability Integral Method (PIM) for goaf location and mining subsidence monitoring, providing a new approach for mining area subsidence monitoring [27]. Leveraging its extensive coverage, InSAR enables area-based monitoring of subsidence in mining regions. This technology is particularly sensitive to minor deformations, allowing for the localization of subsidence basins and monitoring of subsidence boundaries [28,29]. Therefore, building upon the geological and mining conditions of a specific mining panel as the engineering context, this study aims to conduct research on monitoring methods, parameter inversion, and applications for mining-induced surface subsidence in mountainous areas. This is achieved by synergistically utilizing monitoring data from PPP and InSAR technologies.

## 2. Materials and Methods

### 2.1. Study Area

The mine under investigation belongs to the Shanxi Xishan Coal and Electricity Power Company Limited. Its mining field is situated at the junction between the eastern flank of the Guandi Mountains in the Lüliang Mountain range and the Yunzhong Mountains, characterized as a low-to-medium relief mountainous region, with coal resources featuring shallow burial depths and thick seams. The terrain in the study area is relatively complex, with bedrock exposed in most areas, except for loess covering some hilltops. The general topography slopes from higher elevations in the southwest to lower elevations in the northeast. The lowest point is located at the Fen River bed in the northeastern part of the mining field, with an elevation of approximately 1000 m. The highest point is near Borehole No. 531 at the southwestern corner, with an elevation of 1305.35 m. The relative relief within the study area typically ranges from 150 to 250 m. Currently, mining panel 22618 is under active extraction. A conveyor belt corridor and its drive station are in operation on the surface approximately 1300 m from the open-off cut of this panel. According to general principles of mining subsidence, extraction from this panel is expected to induce subsidence and potential damage in the area extending to the conveyor belt corridor and its drive station. Although some knowledge exists regarding surface subsidence patterns induced by mining in shallow coal seams, the current mining depths in this mine are relatively greater, rendering these existing patterns not fully applicable. Therefore, a specific study and analysis of mining-induced surface subsidence for this mine is required.

**Figure 1:**
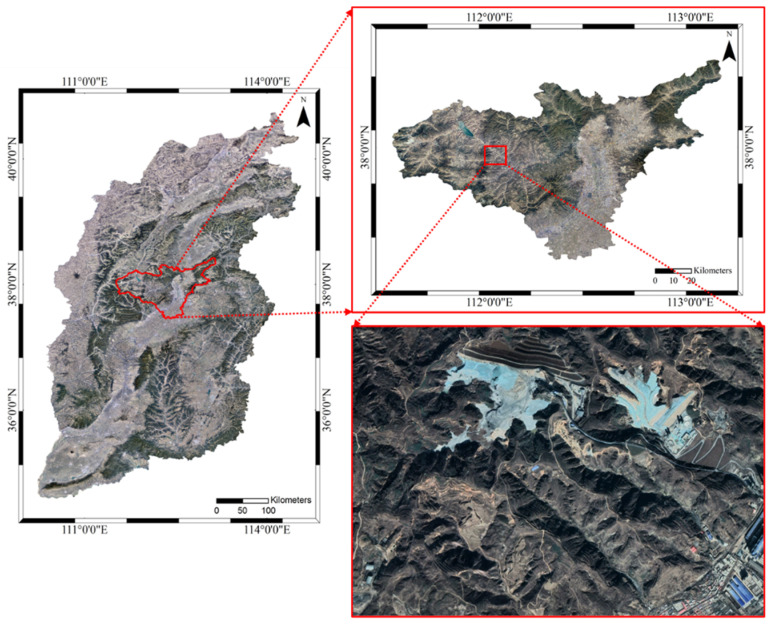
Geographical location and extent of the study area.

Panel 22618 was selected as the study area, and its geographical location and extent are shown in Figure 1. Underground, Panel 22618 is located in the South Sixth Mining District. It borders Panel 222620 to the east, the mine boundary to the south, and the 760-East First Main Return Airway, Conveyor Belt Roadway, and Track Roadway to the north. The remaining surrounding areas are unmined. The coal seam being mined in Panel 22618 is Seam No. 2.3, with a thickness ranging from 2.7 to 3.85 m (classified as medium-thick to thick seam), averaging 3.40 m. The average dip angle of the seam is 4°. The panel has a strike length of 2092 m and a dip width of 180 m. The surface elevation above the panel ranges from 1135 to 1250 m, while the panel’s elevation ranges from 721 to 800 m. Extraction is conducted using comprehensive mechanized mining.

This study focuses on the spatial distribution and patterns of surface subsidence during mineral extraction in Panel 22618. Sentinel-1A imagery covering the entire panel was selected for monitoring due to its open-access policy, stable revisit cycle, and global coverage capabilities. The primary dataset includes 35 ascending Sentinel-1A scenes acquired from April 2016 to September 2017, with “VV” polarization, a radar wavelength of 5.6 cm, and a spatial resolution of 5 m (range) by 20 m (azimuth). A 30 m resolution SRTM DEM and precise orbit files for the Sentinel-1A satellite were also utilized to enhance the accuracy of the InSAR monitoring results.

**Figure 2:**
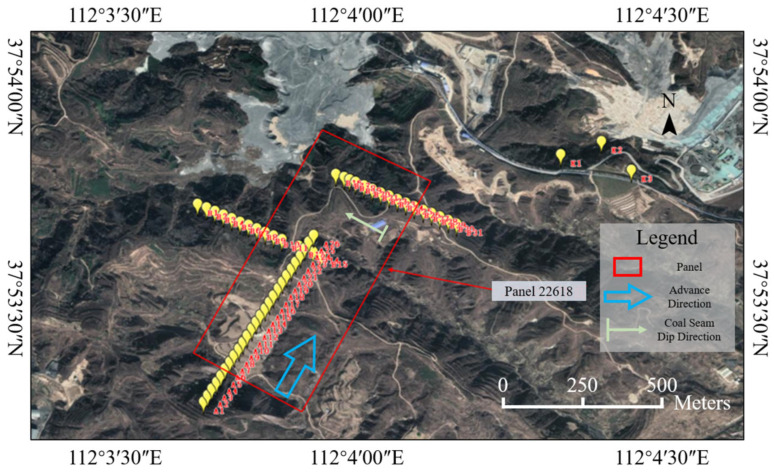
Distribution of the GNSS monitoring network over mining panel 22618.

Considering the geological and geomorphological conditions above the mining area of Panel 22618 and the correspondence between the surface and underground workings, and adhering to the principles of long-term effectiveness and reliability of measurements, three GNSS observation lines were established on the surface above the panel, with a spacing of 30 m between observation points. The spatial relationship between the observation points and Panel 22618 is shown in Figure 2. One line is arranged along the strike direction, extending from the open-off cut end to the main section, comprising 26 monitoring points. The other two lines are arranged along the dip direction, located on the left and right sides of the fully mined area within the panel, totaling 31 monitoring points. To better understand the impact of mining on surface subsidence, an additional 21 monitoring points were deployed along the conveyor belt corridor and towards the drive station.

In accordance with the observational requirements for surface mining subsidence specified in the Coal Mine Surveying Regulations, all monitoring points were constructed using prism-shaped concrete markers with cross-marked steel bars as central benchmarks. These markers are designed to remain reliably preserved and firmly bonded to the ground surface throughout the monitoring period. Daily observations were conducted in static positioning mode, with continuous data collection at each monitoring point. The single-session data acquisition time was generally no less than 50 min to ensure positioning accuracy. The observation campaign was carried out from 8 April to 9 April 2016, conducting a comprehensive survey of surface mining subsidence above Panel 22618. Subsequently, with the advance of the mining face, a total of ten observations of surface movement and deformation were conducted, with the specific dates listed in Table 1.

### 2.2. Methods

#### 2.2.1. Monitoring of Mining-Induced Surface Subsidence Based on PPP Technology

Conventional surface monitoring networks for mining subsidence in mountainous areas often deviate from standard layout designs due to challenging terrain. Furthermore, relative positioning techniques typically require connection to high-precision known control points. The complex topography of mountainous regions makes it difficult and costly to establish a sufficiently dense network of observation points, significantly increasing both the difficulty and expense of surface subsidence monitoring. In response to these challenges, researchers from universities and institutions worldwide have jointly advanced the development of technical solutions for PPP data processing methods. This technique enables the continuous, rapid, and high-precision acquisition of surface deformation data, providing critical support for monitoring, assessing, and issuing early warnings regarding mining safety and geological health [30,31]. PPP technology achieves precise positioning using only a single dual-frequency GNSS receiver, eliminating the need for connection to reference stations or high-order control points. This capability facilitates the establishment of a seamless, globally unified, high-precision positioning service framework. It offers a practical solution for control surveying in topographically challenging areas, simultaneously reducing labor, material costs, and operational timelines, thereby significantly improving surveying efficiency. PPP has been widely applied in monitoring large-scale regional subsidence and infrastructure safety, including applications in landslide monitoring, seismic deformation studies, and infrastructure positioning [32,33]. Currently, mainstream monitoring techniques in mining areas primarily rely on post-processing precise positioning methods based on GPS-only or GPS + QZSS systems, such as Real-Time Kinematic (RTK) or Continuously Operating Reference Station (CORS) networks. In contrast, the BeiDou Navigation Satellite System (BDS) PPP technology—developed independently in China, offering unrestricted service coverage, enhanced security, real-time capability, low cost, and high reliability—has not yet seen widespread application in mining area surface monitoring. Therefore, comprehensively considering factors such as topography, monitoring network geometry, cost, and operational security, this study optimized the conventional static GPS surveying method. We propose an enhanced monitoring approach based on a static GNSS dual-reference-station methodology to conduct the relevant monitoring tasks.

Given that existing ionospheric delay models offer limited accuracy and the parameters for a functional model are difficult to estimate reliably, a common practice is to form ionosphere-free linear combinations using dual-frequency carrier-phase and pseudorange observations. These new combined observations serve as the functional model. This approach is widely adopted in various orbit determination and positioning systems or software due to its effectiveness in eliminating most first-order ionospheric delay effects and its model simplicity. However, this combination increases the measurement noise by approximately a factor of three and causes the ambiguity parameters to lose their integer nature, leading to prolonged ambiguity convergence time [34]. The ionosphere-free linear combinations for pseudorange and carrier phase observations can be respectively expressed as

(1) $$P_{c}=\frac{f_{1}^{2}}{f_{1}^{2}-f_{2}^{2}}P_{1}-\frac{f_{2}^{2}}{f_{1}^{2}-f_{2}^{2}}P_{2}$$

(2) $$\Phi_{c}=\frac{f_{1}^{2}}{f_{1}^{2}-f_{2}^{2}}\Phi_{1}-\frac{f_{2}^{2}}{f_{1}^{2}-f_{2}^{2}}\Phi_{2}$$

In the equations above, $P_{c}$ and $\Phi_{c}$ denote the ionosphere-free linear combinations of pseudorange and carrier-phase observations, respectively; $P_{1}$ and $P_{2}\;$ represent the pseudorange observations at frequencies $f_{1}$ and $f_{2}$; $\Phi_{1}$ and $\Phi_{2}$ represent the carrier-phase observations at frequencies $f_{1}$ and $f_{2}$.

Prior to conducting PPP resolution, it is first necessary to define the stochastic model for error allocation, which characterizes the random properties of the parameters to be estimated from the observations. Common methods for determining the stochastic model in PPP include the satellite elevation angle method, the signal-to-noise ratio (SNR) method, and the variance component estimation method, among others. Among these, the satellite elevation angle-based method and its variants are the most widely applied for stochastic model determination. This method expresses the observation noise σ as a function of the satellite elevation angle E, with its mathematical representation given by

(3) $$\sigma^{2}=a^{2}+b^{2}\cos^{2}E$$

#### 2.2.2. SBAS-InSAR Methodology for Deformation Monitoring

The SBAS-InSAR technique was developed on the foundation of D-InSAR. Prior to interferometric processing, multiple SAR images covering the same study area are grouped into several subsets based on predefined spatial and temporal baseline thresholds. This grouping strategy aims to maintain high coherence in the generated interferograms, thereby mitigating the impacts of temporal-spatial decorrelation and atmospheric delays [35,36]. When solving for deformation rates, this method addresses the rank deficiency in the system of equations through Singular Value Decomposition (SVD), ultimately obtaining the minimum-norm least squares solution for the deformation velocity field.

Assuming that a total of N + 1 SAR images covers the study area (ordered by acquisition time as t_0_, t_1_, t_2_, …, t_n_), one image is selected as the super master. By performing interferometric pairing between this super master and each of the other N images to generate differential interferograms, a set of M interferometric pairs is obtained, where M satisfies the condition

(4) (N+1)/2≤M≤N(N+1)/2

For any selected interferometric pair, the phase value at a high-coherence point (*x, r*) in the i-th differential interferogram, generated from SAR images acquired at slave time *t_A_* and master time *t_B_* (where *t_B_ > t_A_*), can be expressed as

(5) $$\begin{array}{cc} \delta \Phi_{j} & =\Phi_{B}\left(x,r\right)-\Phi_{A}\left(x,r\right)\;\\ & \approx \frac{4\pi }{\lambda }\left[d\left(t_{B},x,r\right)-d\left(t_{A},x,r\right)\right]+∆\varphi_{i}^{topo}\left(x,r\right)+∆\varphi_{i}^{res}\left(x,r\right) \end{array}$$

In the above equation, $\phi_{A}(x,r)$ and $\phi_{B}(x,r)$ represent the deformation phases at pixel (*x, r*) at times $t_{A}\;$ and $t_{B}$, respectively; j∈(1, 2,…,M); λ denotes the radar wavelength; d denotes the line-of-sight (LOS) deformation; $∆\varphi_{i}^{topo}\left(x,r\right)$ stands for the topographic phase residual; and $∆\varphi_{i}^{res}\left(x,r\right)$ denotes the residual phase term. Consequently, the average LOS deformation velocity $v_{i}$ between times $t_{A}$ and $t_{B}$ can be expressed as

(6) $$v_{i}=\frac{d(t_{B},x,r)-d(t_{A},x,r)}{t_{B}-t_{A}}$$

The phase of the i-th differential interferogram can therefore be expressed as the integral of the deformation rate over the time interval between its master and slave acquisitions. This relationship can be formulated in matrix notation as

(7) Βυ=δϕ

Due to the multi-master acquisition strategy employed by SBAS-InSAR, the design matrix Β is often rank-deficient. Consequently, the SVD method is utilized to solve the system of equations, yielding the minimum-norm least squares solution for the deformation rate vector υ. Subsequently, by integrating the deformation rates over each time interval, the cumulative deformation throughout the entire observation period can be obtained.

#### 2.2.3. Integrated InSAR-GNSS Monitoring of Mining-Induced Surface Subsidence

InSAR is an effective means for obtaining surface subsidence information, capable of accurately capturing the deformation patterns at the edges of subsidence basins. However, due to the inherent limitations of SAR imagery and the influence of various noise and error sources, this technique often fails to reliably detect true and precise subsidence information in the central regions of mining areas, where deformation gradients are high. Consequently, InSAR-derived results in these areas can deviate significantly from actual conditions. Conversely, GNSS monitoring technology offers high precision in key subsidence zones. Nevertheless, its application is constrained by factors such as topography and cost, making it difficult to achieve comprehensive area-wide monitoring. This limitation often results in reduced monitoring accuracy at the margins of the subsidence basin. By integrating the respective strengths of InSAR and GNSS technologies for subsidence monitoring, it is possible to acquire complete and accurate surface deformation information across the entire mining area. The specific procedural steps are as follows:

(1) A set of N + 1 SAR images covering the mining area is selected. The SBAS-InSAR technique is applied to this dataset for processing and analysis to obtain an initial estimate of the deformation field across the mining region.

(2) Relevant field data from the mining area and GNSS monitoring data collected from the mining panel are gathered. These data are then analyzed and processed to derive precise subsidence information, particularly for the central region of the surface subsidence basin.

(3) High-coherence points from the edges of the InSAR-derived subsidence basin are selected, along with GNSS monitoring points located near the subsidence center. These points serve as control or characteristic points. They are then projected into a unified mining-panel coordinate system. Subsequently, a PIM subsidence model for the mining area is established using these integrated data points. This model is finally applied to generate a continuous, spatially complete probability-integral-based subsidence field.

Since the PIM is not a conventional function and lacks a dedicated surface-fitting model, a multivariate function f must be constructed based on this prediction method to perform PIM-based surface fitting. Following the principle of least squares, the optimal fit is achieved when the value of f is minimized; the parameters determined under this condition represent the optimal fitting parameters between the PIM and the measured data. The PIM model W and the constructed multivariate function f are given in Equations (8) and (9) [37,38].

(8) $$\begin{array}{cc} W\left(x,y\right)= & \frac{1}{4}mq\cos\alpha \left[erf\left(\frac{\sqrt{\pi }\tan\beta_{3}}{H_{0}\left(1+\tan k\;\alpha \tan\alpha \right)}x\right)-erf\left(\frac{\sqrt{\pi }\tan\beta_{3}}{H_{0}\left(1+\tan k\;\alpha \tan\alpha \right)}\left(x-\left(L_{3}-2s_{3}\right)\right)\right)\right]\\ & \left[erf\left(\frac{\sqrt{\pi }\tan\beta_{1}}{H_{1}}y\right)-erf\left(\frac{\sqrt{\pi }\tan\beta_{2}}{H_{2}}\left(y-\left(L_{1}-s_{1}-s_{2}\right)\right)\frac{\cos\left(1-k\right)\alpha }{\cos k\;\alpha }\right)\right] \end{array}$$

In the equation: $\left(x,y\right)$ are the coordinates of an arbitrary surface point; q is the subsidence factor; α is the dip angle of the coal seam; $H_{0}$ is the mining depth at the center of the panel; $H_{1}$ is the mining depth at the downhill boundary of the panel; $H_{2}$ is the mining depth at the uphill boundary of the panel; $L_{1}$ is the extraction width along the dip direction; $L_{3}$ is the extraction width along the strike direction; $\tan\beta_{1}$ is the tangent of the main influence angle for the uphill side; $\tan\beta_{2}$ is the tangent of the main influence angle for the downhill side; $\tan\beta_{3}$ is the tangent of the main influence angle for the strike direction; $s_{1}$ is the inflection point offset for the downhill side; $s_{2}$ is the inflection point offset for the uphill side; $s_{3}$ is the inflection point offset for the strike direction; and k is the propagation angle coefficient of mining-induced subsidence influence.

(9) $$f\left(q,\tan\beta_{1},\tan\beta_{2},\tan\beta_{3},s_{1},s_{2},s_{3},k\right)=\sum_{i=1}^{n}\omega_{i}{\left[W\left(x,y\right)-z_{i}\right]}^{2}=min$$

In the equation: $\omega_{i}$ is the weight function, with a default value of 1; $z_{i}$ is the measured surface subsidence value.

### 2.3. Accuracy Analysis of PPP Monitoring

Due to mining impacts, the national high-order control points surrounding the mining area have suffered significant damage. Consequently, current surface subsidence monitoring and related engineering activities in the area primarily rely on a CORS network established within the mining area. The station names are B23, A12, and A10, respectively. PPP processing was performed for these CORS stations, and the statistical information on their positioning accuracy is shown in Table 2.

As can be seen from the table above, the convergence in the N direction is the fastest, achieving centimeter-level positioning accuracy within just 1 h. This level of accuracy meets the precision requirements for real-time monitoring of mine surface deformation, such as in the monitoring of open-pit mine slopes. Once the ambiguities are successfully converged and fixed, high-precision, real-time, and automated monitoring and early warning of surface deformation can be achieved.

**Figure 3:**
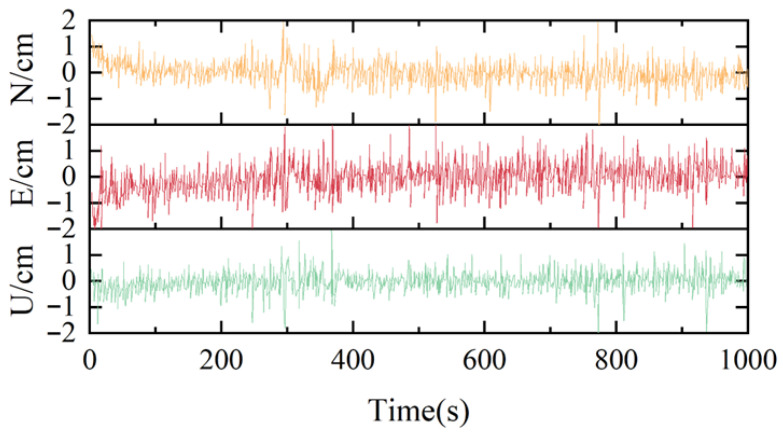
Time-series solution results for Point A6 using Scheme 1.

**Figure 4:**
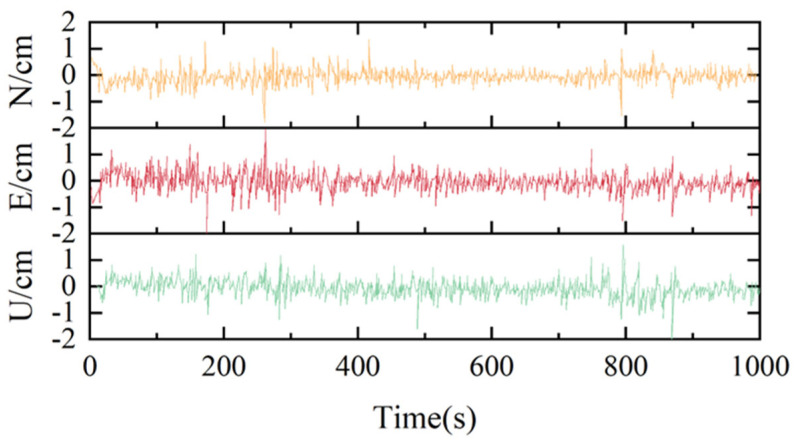
Time-series solution results for Point A6 using Scheme 2.

To validate the effectiveness of the algorithm proposed in this study, GNSS measurement data from several dozen monitoring points in the mining area were utilized. Taking monitoring point A6 as an example, an analysis of PPP time-series monitoring accuracy was conducted. Concurrently, two distinct data processing schemes employing different filtering algorithms were designed: Scheme 1 utilizes the conventional Kalman filter, while Scheme 2 employs a Kalman filter based on an adaptive factor. Figure 3 and Figure 4 respectively present the positioning errors for monitoring point A6 at each epoch under the two processing schemes.

By comparing the positioning errors in each direction shown in Figure 3 and Figure 4, it can be observed that the error amplitude of Scheme 2 is significantly smaller than that of Scheme 1. This demonstrates that the Kalman filter algorithm based on an adaptive factor plays a crucial role, effectively mitigating the influence of prior model bias and improving positioning accuracy. However, sporadic outliers still exist in certain epochs, where the solution accuracy remains low. This situation is typically caused by abnormal observations, a problem that neither the conventional Kalman filter nor the adaptive-factor-based Kalman filter can effectively resolve. In the horizontal directions, the positioning accuracy for the vast majority of epochs is within 1 cm. Compared to the horizontal accuracy, the vertical accuracy is lower, yet it remains within 2 cm for most epochs. For large-scale monitoring of surface deformation in mining areas, this level of accuracy provides effective monitoring information and delivers essential data support for safety forecasting and early warning in mining regions.

**Figure 5:**
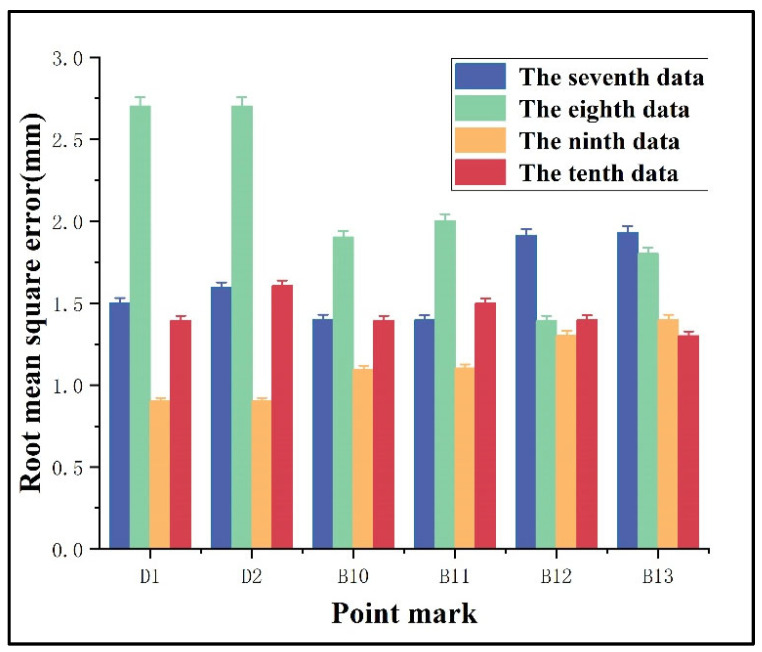
RMSE of the computed elevations for the reference points and selected monitoring points.

Given that the horizontal accuracy of GNSS measurements is superior to their vertical accuracy, this study focuses solely on the analysis of elevation measurement precision to validate the proposed monitoring method for mining-induced surface subsidence in mountainous areas. Taking the data from epochs 7, 8, 9, and 10 for two reference points (D1, D2) and monitoring points (B10, B11, B12, B13) as an example, the distribution of the root mean square error (RMSE) in the solutions, derived from relevant data processing, is illustrated in Figure 5. The results from the reference points and selected monitoring points indicate that the maximum RMSE in elevation is 2.7 mm, meeting the precision requirements for monitoring surface mining subsidence. Under the influence of Earth’s curvature, slight variations exist in the computed elevations of the reference points across different epochs. The elevations of the reference points and the elevation differences between two consecutive epochs are presented in Table 3. These results confirm the correctness of the observational data for each epoch and demonstrate the reliability of the monitoring method proposed in this study.

## 3. Results

### 3.1. GNSS Monitoring Results

**Figure 6:**
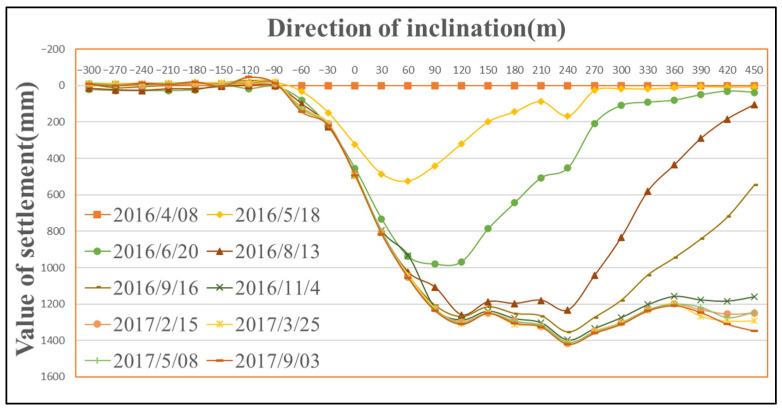
Dynamic surface subsidence along the strike observation line.

**Figure 7:**
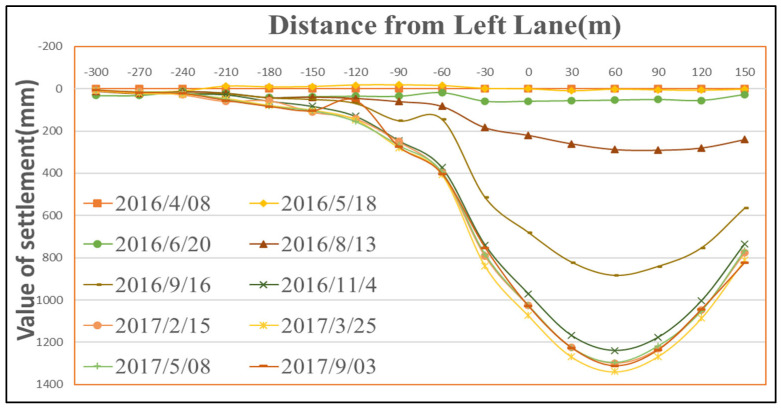
Dynamic surface subsidence along the left-dip observation line of the mining panel.

**Figure 8:**
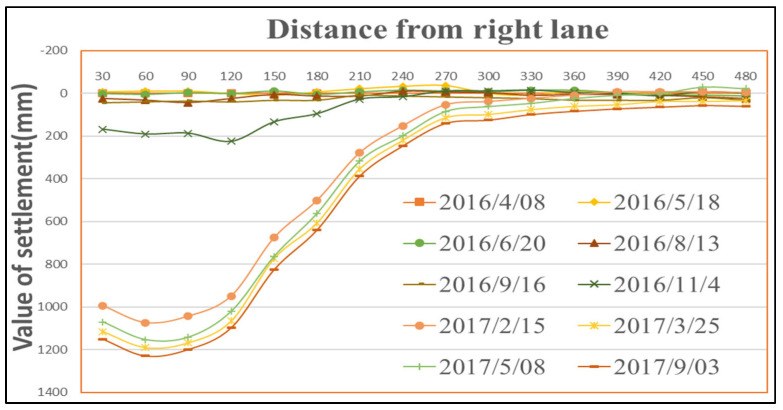
Dynamic surface subsidence along the right-dip observation line of the mining panel.

In accordance with the observation requirements for surface mining subsidence stipulated in coal mine surveying regulations, this study conducted a total of 10 surface subsidence monitoring campaigns at Panel 22618 between 8 April 2016 and 3 September 2017. Data processing was performed using the open-source software MG-APP 2.0.8, which incorporates multiple tropospheric models and estimation algorithms [39]. Figure 6, Figure 7 and Figure 8 present the dynamic surface subsidence curves observed along the strike observation line, the left-dip observation line, and the right-dip observation line of the panel, respectively. As shown in Figure 6, the point of maximum surface subsidence is A19, with a maximum subsidence of 1438 mm. By 3 September 2017, the surface subsidence had stabilized. In the central part of the subsidence basin, the subsidence curve is not flat-bottomed, primarily due to the influence of mountainous terrain. Point A19 is located at the toe of a near-vertical slope, an area prone to water scour. Under the combined effects of collapsible loess and mining activities, its surface subsidence is slightly greater than that of the adjacent point A18. Similarly, points A20 and A21, situated on a slope, exhibit slightly larger subsidence values due to the influence of slope creep.

(1) Angle of Full Subsidence

From the analysis above, under conditions of full subsidence, the horizontal projection distance from the edge of the flat bottom of the surface movement basin on the main section to the open-off cut is 240 m, while the mining depth is 430 m. Therefore, according to the definition of the angle of full subsidence, the strike angle of full subsidence is calculated as $\psi_{3}=arctan(430/240)=61{}^{\circ}$.

(2) Angle of Maximum Subsidence

The angle of maximum subsidence θ is defined on the inclined main section as the angle measured on the downhill side of the coal seam between the horizontal line and the line connecting the midpoint of the goaf with the surface projection of the point of maximum subsidence in the surface movement basin.

As shown in Figure 7 and Figure 8, the point of maximum subsidence along the left-dip observation line is B13, located 60 m from the downhill boundary. Similarly, the point of maximum subsidence along the right-dip observation line is B17, also situated 60 m from the downhill boundary. Therefore, it can be concluded that the point of maximum surface subsidence for Panel 22618 is located approximately 60 m from the downhill boundary. Given that the dip width of the panel is 180 m, the angle of maximum subsidence is calculated as θ=arctan(430/30)=86°.

(3) Advance Influence Angle and Advance Influence Distance

Based on the strike monitoring data, the advance influence distance l is determined to be 150 m. Given that the average mining depth $H_{0}$ of Panel 22618 is 430 m, the advance influence angle is calculated as $\omega =\mathrm{arccot}(l/H_{0})=71{}^{\circ}$.

(4) Lag Distance of Maximum Subsidence Velocity and its Lag Angle

**Figure 9:**
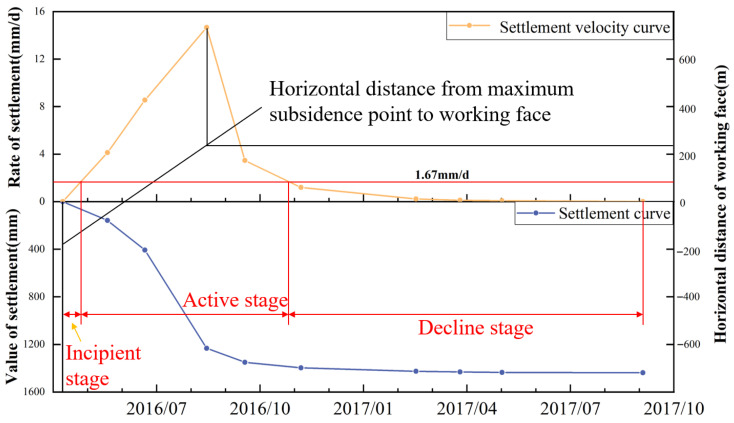
Subsidence velocity and cumulative subsidence curve at the surface point of maximum subsidence.

As shown in Figure 6, the surface point of maximum subsidence is A19. Based on the monitoring data from point A19, the subsidence velocity and cumulative subsidence curve for this point were plotted, as presented in Figure 9. As the mining panel advanced, the relative position between the point of maximum subsidence velocity and the working face remained essentially constant, with the point of maximum velocity advancing in a regular pattern. It can be observed that once full subsidence was reached at the surface, the point of maximum subsidence velocity on the velocity curve consistently lagged behind the advancing working face by a fixed distance. This fixed distance is defined as the lag distance of maximum subsidence velocity L, measured as 228 m. The angle between the line connecting the point of maximum subsidence velocity and its corresponding working face position and the seam horizon (horizontal line) on the goaf side is termed the lag angle of maximum subsidence velocity, denoted by Φ. It is calculated as $\Phi =arctan(H_{0}/L)=62^{\circ }$.

Furthermore, Figure 9 clearly shows that the maximum subsidence velocity at the surface point of maximum subsidence is 14.46 mm/d. According to general conventions in mining subsidence science, the critical value for determining whether surface subsidence is in an active phase is 50 mm/month. Analysis of the measured data from the point of maximum subsidence along the strike observation line (as shown in Figure 9) indicates that the active period for this point lasted from 24 April 2016 to 25 October 2016, spanning a duration of 184 days.

(5) Limit Angle

According to the definition of the limit angle, under conditions of full or nearly full subsidence, the angle measured on the pillar side between the horizontal line and the line connecting the boundary point of the surface movement basin on the main section to the edge of the goaf is termed the limit angle. Analysis of the strike observation line data reveals that from the initial observation until 13 August 2016, surface monitoring points outside the open-off cut experienced subsidence. Beginning on 16 September 2016, these points started to exhibit uplift. Therefore, the monitoring data from 13 August 2016 were selected for determining the limit angles of Panel 22618. This dataset shows that the surface subsidence near the open-off cut closely approximates the subsidence expected under full extraction. Consequently, the strike limit angle can be calculated as $\delta_{0}=arctan(430/150)=71{}^{\circ}$. Based on the left-dip observation line data, the downhill limit angle is $\beta_{0}=arctan(430/300)=55{}^{\circ}$. Similarly, using the right-dip observation line data, the uphill limit angle is $\gamma_{0}=arctan(430/210)=64{}^{\circ}$.

(6) Angle of Critical Deformation

According to the definition of the angle of critical deformation, under conditions of full or nearly full subsidence, the angle measured on the pillar side between the horizontal line and the line connecting the outermost point among the three critical deformation values on the main section of the surface movement basin to the edge of the goaf is termed the angle of critical deformation.

By compiling the strike observation data, the patterns of surface tilt, curvature, and horizontal strain along the strike observation line can be derived. The analysis shows that the maximum positive surface tilt is 10.5 mm/m and the maximum negative surface tilt is −8.5 mm/m. The point where the surface tilt equals 3 mm/m is located 90 m outside the open-off cut. The maximum positive surface curvature is 0.23 mm/m^2^ and the maximum negative surface curvature is −0.27 mm/m^2^. The point where the surface curvature equals 0.2 mm/m^2^ is situated approximately 40 m outside the open-off cut. The maximum positive horizontal strain is 5 mm/m, and the maximum negative horizontal strain is −4 mm/m. The point where the horizontal strain equals 2 mm/m is located 75 m outside the open-off cut. Synthesizing the above data for the strike direction, the angle of critical deformation (movement angle) is calculated as δ=arctan(430/90)=78°.

By compiling the left-dip observation line data, the patterns of surface tilt, curvature, and horizontal strain along this line can be derived. The analysis indicates that the maximum positive surface tilt is 14.5 mm/m and the maximum negative surface tilt is −9.6 mm/m. The point where the surface tilt equals 3 mm/m is located 110 m outside the downhill boundary. The maximum positive surface curvature is 0.42 mm/m^2^ and the maximum negative surface curvature is −0.23 mm/m^2^. The point where the surface curvature equals 0.2 mm/m^2^ is situated approximately 75 m outside the downhill boundary. The maximum positive horizontal strain is 5.9 mm/m and the maximum negative horizontal strain is −5.5 mm/m. The point where the horizontal strain equals 2 mm/m is located 120 m outside the downhill boundary. Synthesizing the above data for the downhill direction, the angle of critical deformation is calculated as β=arctan(430/120)=74°.

Based on the right-dip observation line data, the patterns of surface tilt, curvature, and horizontal strain along this line can be obtained. The analysis shows that the maximum positive surface tilt is 2.8 mm/m and the maximum negative surface tilt is −9.7 mm/m. The point where the surface tilt equals 3 mm/m is located 80 m outside the uphill boundary. The maximum positive surface curvature is 0.14 mm/m^2^ and the maximum negative surface curvature is −0.21 mm/m^2^. The point where the surface curvature equals 0.2 mm/m^2^ is situated within the mining panel. The maximum positive horizontal strain is 2.3 mm/m and the maximum negative horizontal strain is −8.1 mm/m. The point where the horizontal strain equals 2 mm/m is located 80 m outside the uphill boundary. Synthesizing the above data, the angle of critical deformation (movement angle) for the uphill direction is calculated as γ=arctan(430/80)=79°.

(7) Probability-Integrated Subsidence Prediction Basin

**Figure 10:**
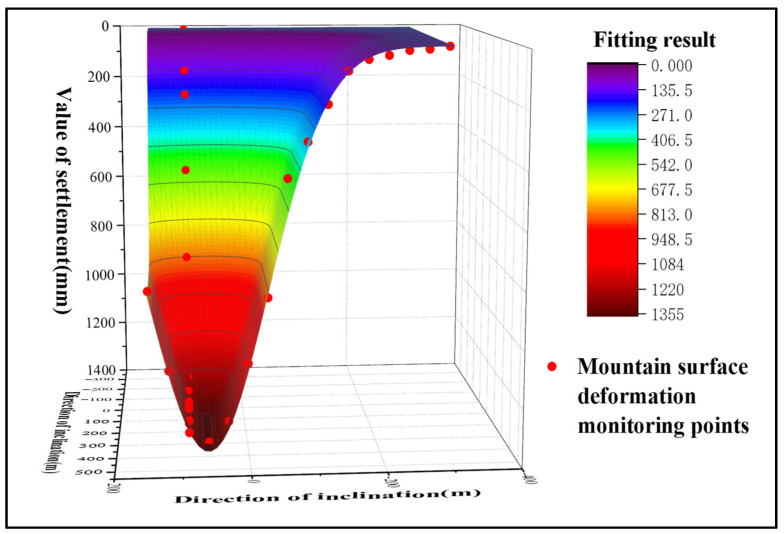
3D Visualization of the Mining Area Subsidence Prediction Basin Derived from GNSS Probability-Integrated Monitoring.

By substituting the GNSS monitoring results into the probability-integral fitting function, a continuous ground subsidence integral model was obtained. The result is presented in Figure 10. It can be observed that during the study period, a distinct funnel-shaped subsidence basin developed above Panel 22618, as detected by GNSS technology. However, this method indicated almost no subsidence around the periphery of the panel, with the subsidence basin converging rapidly at its edges. The reason for this discrepancy is that in reality, mining-induced subsidence is often influenced by complex topography, groundwater loss, and extraction from other goafs. In contrast, the PIM considers only ground subsidence caused by the mining activity itself, resulting in the predicted subsidence being predominantly concentrated directly above the mining panel.

### 3.2. Mining Area Subsidence Monitoring via InSAR

**Figure 11:**
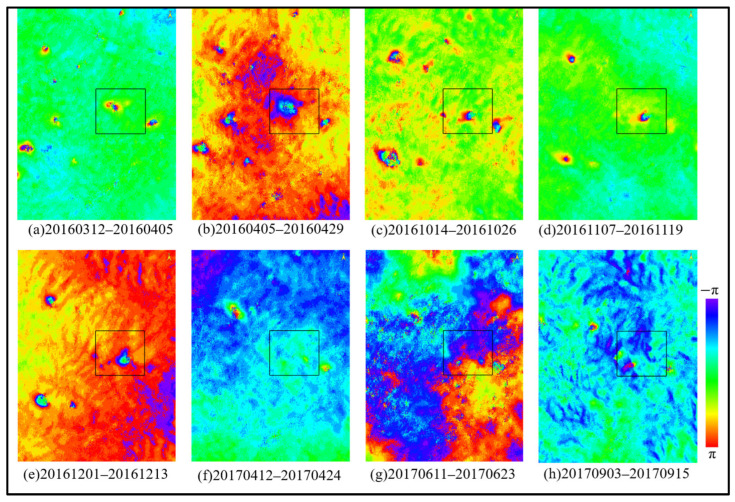
Example of the Filtered Differential Interferogram. (**a**) 2016.03.12–2016.04.05; (**b**) 2016.04.05–2016.04.29; (**c**) 2016.10.14–2016.10.26; (**d**) 2016.11.07–2016.11.19; (**e**) 2016.12.01–2016.12.13; (**f**) 2017.04.12–2017.04.24; (**g**) 2017.06.11–2017.06.23; (**h**) 2017.09.03–2017.09.15.

**Figure 12:**
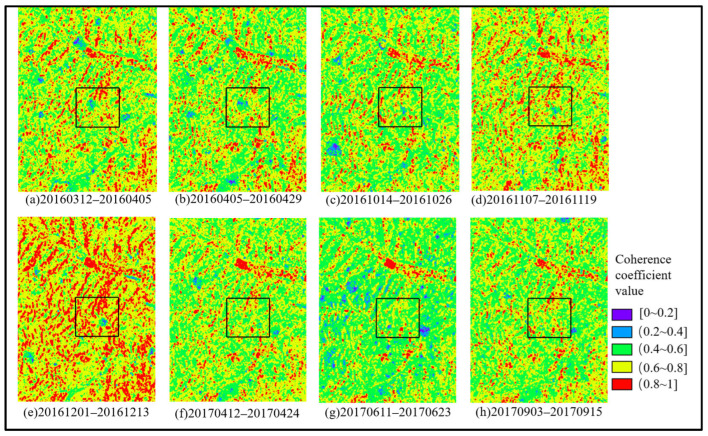
Example of the Coherence Coefficient Map. (**a**) 2016.03.12–2016.04.05; (**b**) 2016.04.05–2016.04.29; (**c**) 2016.10.14–2016.10.26; (**d**) 2016.11.07–2016.11.19; (**e**) 2016.12.01–2016.12.13; (**f**) 2017.04.12–2017.04.24; (**g**) 2017.06.11–2017.06.23; (**h**) 2017.09.03–2017.09.15.

The generated interferometric pairs were subjected to sequential differential processing in chronological order. Considering that the interferometric phase is inevitably affected by various noise sources, an adaptive filtering method was employed for noise removal. This process yielded enhanced differential interferometric phases and their corresponding coherence coefficient maps, as shown in Figure 11 and Figure 12, respectively.

As shown in Figure 11, during the initial monitoring period in the mining area, interference fringes were sparse. Subsequently, over time, the fringes within the rectangular area became increasingly dense, leading to the conclusion that this area likely experienced surface subsidence during the study period. Correlating with Figure 12, it is evident that during the monitoring periods corresponding to interferometric pairs Figure 12b–e, coherence was relatively high, and the corresponding differential interferograms exhibit clear and complete interference fringes. In contrast, interferometric pairs Figure 12f,g are subject to greater noise and severe decorrelation. This is attributed to higher vegetation coverage in summer or excessive deformation gradients that exceed the practical monitoring capability of the imagery. Comparing the differential interferograms from these periods, the interference fringes in the subsidence areas appear disordered and even fail to form complete fringe patterns.

**Figure 13:**
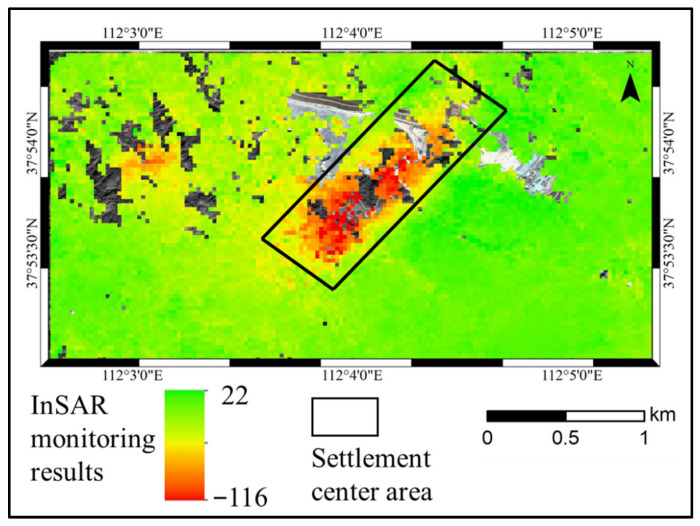
Results of InSAR-based Subsidence Monitoring.

As shown in Figure 13, the location and extent of subsidence detected by InSAR correspond with the actual mining panel in the study area. However, InSAR exhibits a significant deficiency in monitoring large-gradient deformation within the central subsidence zone. As mining activities progressed, subsidence in the area continued to increase. Leveling measurements recorded a maximum cumulative subsidence of 1438 mm over the panel, whereas the maximum subsidence detected by InSAR was only 116 mm, indicating a substantial discrepancy from the actual conditions in the mining area.

### 3.3. Integrated Monitoring Results from InSAR and GNSS

**Figure 14:**
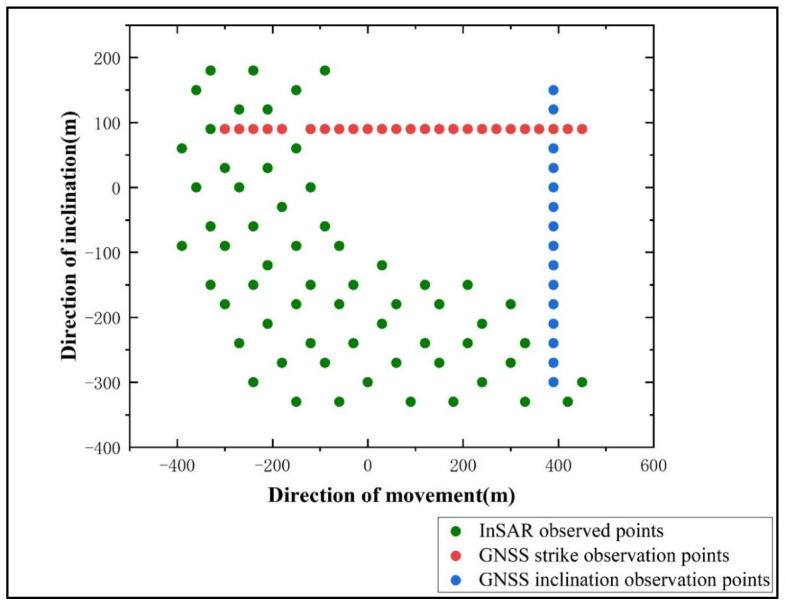
Distribution of Feature Points.

To construct the Probability Integral Model, high-coherence feature points were further extracted from the InSAR-derived subsidence results. For Sentinel-1A SAR imagery, InSAR monitoring results are considered unreliable when the coherence coefficient is less than 0.3. In practical applications, due to the characteristics of mining subsidence, such as rapid deformation rates and large gradients, the monitoring accuracy imposes more stringent requirements on coherence. Therefore, based on the coherence conditions in the mining area depicted in Figure 12, a coherence threshold of 0.8 was selected. Feature points with coherence values greater than 0.8 were identified as high-coherence points. These points, along with the GNSS monitoring points, were then transformed into the mining panel coordinate system, as illustrated in Figure 14.

**Figure 15:**
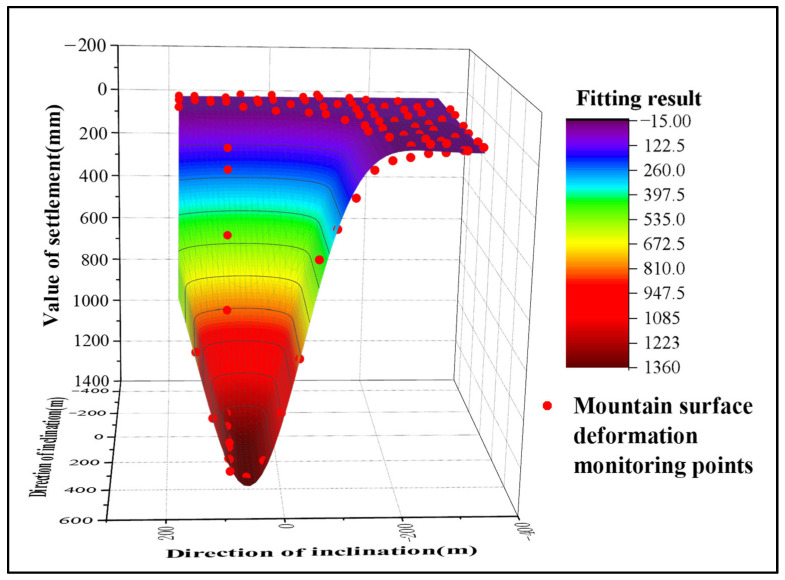
Subsidence Monitoring Results from the Integrated Method.

Using these data as fitting inputs, the InSAR-derived subsidence edges were integrated with the probability-integral-modeled subsidence center. This fusion yielded a complete subsidence basin for the mining area, with the result presented in Figure 15.

As shown in Figure 15, ground subsidence was detected in the mining area by the integrated method between April 2016 and September 2017. The location of the subsidence basin closely aligns with that of Panel 22618. During the study period, the method also detected minor subsidence around the periphery of the panel. This is attributed to the presence of a series of small faults near the working face. The ongoing mining activities disturbed the overlying strata, inducing minor deformations that variably affected the surrounding environment. These findings are consistent with the actual mining progress and geological conditions of the area, indicating that the integrated method outperforms the GNSS Probability Integral Method in monitoring the edges of the subsidence basin. Analysis of the integrated method’s results in the central part of the basin reveals that by September 2017, the subsidence center of Panel 22618 was located approximately 360 m from the starting line of extraction, which matches the actual conditions in the mining area. The maximum cumulative subsidence monitored by this method reached 1370 mm, demonstrating its significantly superior capability in monitoring the subsidence center compared to the InSAR technique alone.

## 4. Discussion

**Figure 16:**
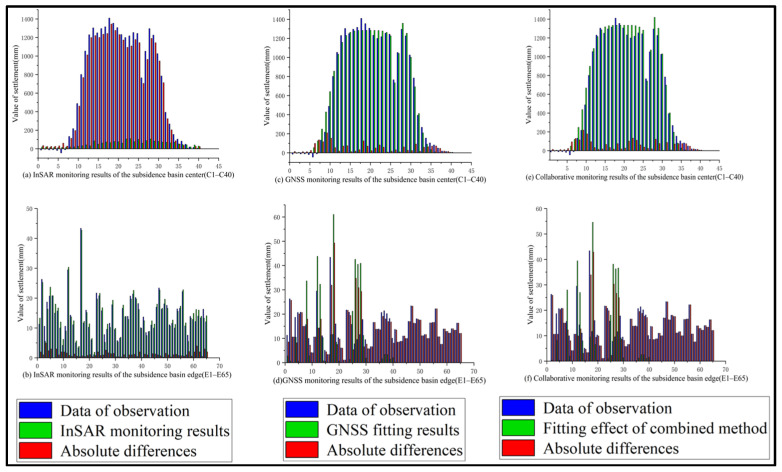
Comparison of Monitoring Results. (**a**) InSAR monitoring results of the subsidence basin center (C1–C40); (**b**) InSAR monitoring results of the subsidence basin edge (E1–E65); (**c**) GNSS monitoring results of the subsidence basin center (C1–C40); (**d**) GNSS monitoring results of the subsidence basin edge (E1–E65); (**e**) Collaborative monitoring results of the subsidence basin center (C1–C40); (**f**) Collaborative monitoring results of the subsidence basin edge (E1–E65).

To further validate the accuracy of the monitoring results obtained by the integrated method, a quantitative comparative analysis was performed on three monitoring approaches: InSAR, the GNSS-based Probability Integral Method, and the Integrated Probability Integral Method. This analysis utilized data from 105 leveling points, which included 40 points (C1–C40) in the central part of the mining subsidence basin and 65 points (E1–E65) at its periphery. The results from these three methods were compared against actual leveling survey data. The comparative outcomes are illustrated in Figure 16, while a statistical comparison of errors among the three methods is provided in Table 4.

Based on the comparative results of the three monitoring methods presented in Figure 16 and Table 4, the following analysis can be drawn:

(1) During the period from April 2016 to September 2017, InSAR, GNSS, and the integrated method all detected ground subsidence above Panel 22618 induced by coal mining operations. The locations and evolving trends of the subsidence basins identified by these methods are largely consistent with each other and align with the leveling survey results.

(2) Compared with the GNSS Probability Integral Method, the integrated method yields results at the edges of the subsidence basin that align more closely with the leveling data. As shown in Figure 10 and Figure 15, the GNSS Probability Integral Method exhibits faster convergence at the basin margins relative to the integrated method. Analysis suggests this is because the Probability Integral Method does not account for external influencing factors during the inversion process, making it less sensitive to deformation at the basin edges. Further examination of the fitting data from both methods reveals that in the marginal areas of the subsidence basin, the integrated method produces monitoring results that better match the actual conditions. Specifically, at the basin edges, the RMSE of the integrated method is 7 mm, representing a 56% improvement in RMSE compared to the Probability Integral Method.

(3) Compared with the InSAR results, the integrated method demonstrates a significant enhancement in monitoring large-gradient deformation in the central subsidence zone. Figure 13 and Figure 15 indicate that the location of the subsidence center detected by InSAR is generally consistent with that identified by the integrated method. However, compared to leveling data, the maximum absolute error of the InSAR results reaches 1345 mm. This discrepancy is primarily due to the large deformation gradient and rapid subsidence rate in the central mining area, which exceed the monitoring threshold of Sentinel-1A imagery. Additionally, factors such as the rugged, dissected terrain and high noise levels in the study area degrade SAR image quality, preventing InSAR from accurately capturing the true subsidence information in the basin center. In the central subsidence zone, the RMSE for the InSAR results and the integrated method results are 814 mm and 17 mm, respectively. Compared to InSAR alone, the integrated method achieves a 97.9% improvement in RMSE.

(4) Based on the error statistics of the three monitoring methods, the RMSE for the entire mining area is 502 mm for InSAR, 45 mm for the GNSS Probability Integral Method, and 16 mm for the integrated method. This indicates an overall improvement in monitoring accuracy compared to both InSAR and the GNSS Probability Integral Method. Specifically, the integrated method achieves a 96.8% reduction in RMSE relative to InSAR and a 64.4% reduction relative to the GNSS Probability Integral Method.

While previous studies have integrated InSAR and GNSS technologies for deformation monitoring, this research distinguishes itself by focusing on the application of this combined approach in mountainous mining areas characterized by complex terrain, high deformation gradients, and limited accessibility—unlike most existing studies that apply fusion techniques in relatively stable or low-gradient deformation environments. The key innovation lies in the introduction of an adaptive fusion method with constraint conditions, which preserves InSAR monitoring results for edge regions with minor deformation, while employing GNSS data for large-scale subsidence central zones and reconstructing them through the probability integral method. This targeted fusion strategy effectively addresses the inherent limitations of InSAR in capturing rapid, large-magnitude subsidence and the sparse spatial resolution of the GNSS. Moreover, by adopting an enhanced PPP processing approach combined with adaptive filtering, the precision of GNSS data in mountainous conditions is significantly improved, thereby enhancing the reliability of the fusion results. Validated through comparative analysis with extensive leveling measurements conducted in both the central and peripheral regions of the subsidence basin, the proposed method not only achieves higher accuracy but also establishes a practical, high-resolution monitoring framework suitable for mine safety management and infrastructure protection in complex terrain areas. Consequently, this study extends the applicability of integrated InSAR-GNSS monitoring technology to more challenging operational scenarios, providing a refined and practically validated workflow with clear implications for advancing sustainable mining monitoring and maintenance practices.

## 5. Conclusions

To address the limitations of traditional GNSS and InSAR technologies in current mining area surface subsidence monitoring, this study focused on Panel 22618 of a coal mine in Shanxi Province. An improved approach for monitoring mining-induced surface subsidence in mountainous areas was developed based on PPP. Building upon this, InSAR monitoring data was integrated to derive the subsidence basin within the mining area during the study period. The main conclusions are as follows:

(1) Using the SBAS-InSAR technique, the ground subsidence rate and cumulative deformation from April 2016 to September 2017 in the study area were obtained. The results indicate that the maximum surface subsidence rate reached −186.67 mm/year, with a total subsidence of up to 248 mm. A large-scale subsidence zone was identified above Panel 22618, developing into an elongated subsidence basin.

(2) By analyzing ten periods of surface observation data from Panel 22618, settlement information along the strike direction and two dip-oriented observation lines were obtained. The maximum subsidence was recorded at observation point A19, with a settlement of 1378 mm. Based on these data, angular parameters and prediction parameters for surface subsidence using the probability integral method under the given geological and mining conditions were inverted, providing technical support and a theoretical basis for evaluating mining-induced damage in the area.

(3) Through the synergistic monitoring of mining subsidence at Panel 22618 using InSAR and GNSS data, the respective advantages of both technologies were fully leveraged. By integrating the two datasets via the probability integral method, a three-dimensional surface deformation field was inverted. The results show that this approach not only reflects the impact of mining in mountainous terrain on surface subsidence near the working face but also accurately captures deformation data in the central subsidence zone. Compared to InSAR-only monitoring and the GNSS-based probability integral method, the proposed method reduced errors by 96.8% and 64.4%, respectively, offering a novel approach for investigating ground subsidence patterns induced by coal mining in mountainous regions.

## Figures and Tables

**Table 1:** GNSS Observation Period in the Study Area.

Number of Observation Sessions	Observation Dates	Number of Observation Sessions	Observation Dates
1	8 April 2016	6	4 November 2016
2	18 May 2016	7	15 February 2017
3	20 June 2016	8	25 March 2017
4	13 August 2016	9	18 May 2017
5	16 September 2016	10	3 September 2017

**Table 2:** Comparison of PPP Accuracy Based on Post-Processed Precise Satellite Products.

Station Point	RMS/cm			STD/cm		
	N	E	U	N	E	U
B23	0.88	1.42	1.98	0.80	0.53	2.76
A12	1.78	2.34	2.29	0.72	0.66	2.35
A10	3.05	2.12	2.28	0.68	0.60	2.16
Average	1.90	1.96	2.18	0.73	0.60	2.42

**Table 3:** Computed elevations of the reference points and elevation differences between two consecutive epochs (unit: mm).

Point ID	Adjusted Elevations from Each Epoch’s Free-Network Adjustment				Elevation Difference		
	Phase 7	Phase 8	Phase 9	Phase 10	7–8	8–9	9–10
D1	1125.244	1124.630	1124.817	1124.711	0.614	−0.187	0.106
D2	1145.474	1144.866	1145.052	1144.948	0.608	−0.186	0.104
D1 − D2	−20.230	−20.236	−20.235	−20.237			

**Table 4:** Error Statistics.

RMSE (mm)	The Edge of Subsidence	The Center of Subsidence	The Overall Results
**InSAR**	6	814	502
**GNSS**	16	70	45
**United method**	7	17	16

## Data Availability

Data are contained within the article.

## Abbreviations

The following abbreviations are used in this manuscript:

GNSS	Global Navigation Satellite System
PPP	Precise Point Positioning
InSAR	Interferometric Synthetic Aperture Radar
D-InSAR	Differential Interferometric Synthetic Aperture Radar
PIM	Probability Integral Method
RTK	Real-Time Kinematic
CORS	Continuously Operating Reference Station
BDS	BeiDou Navigation Satellite System
SNR	signal-to-noise ratio
SVD	Singular Value Decomposition
RSME	root mean square error
